# Does the management of patients with myocardial infarction with nonobstructive coronary arteries (MINOCA) changes with advanced diagnostic workup beyond coronary angiography? Results from the “Evaluation of the clinical Profile, Investigations and Cardiac Imaging of the Patients with MINOCA (EPIC-MINOCA Study)”

**DOI:** 10.1186/s43044-024-00530-1

**Published:** 2024-08-05

**Authors:** Yogesh Chander, Bhanu Duggal, Shishir Soni

**Affiliations:** 1 AIIMS Rishikesh, Rishikesh, India; 2 Super-Specialty Hospital (SSH), NSCB Medical College, Jabalpur, MP India

**Keywords:** MINOCA, Intravascular imaging, Cardiac imaging, Myocardial infarction

## Abstract

**Background:**

Evaluation of the patients with MINOCA and identifying the underlying aetiology remains challenging. However, investigation in most patients remains limited to coronary angiography (CAG). The study aimed to assess the clinical profile, investigations and cardiac imaging of the patients with MINOCA and its outcomes.

**Results:**

Out of 55 patients with MINOCA, CAG was normal in 16 (29.1%), while 39 (69.9%) had nonobstructive coronary artery disease. Of 55 patients, 34 had limited workup (Group 1) and only 21 had advanced workup (Group 2). In comparison to Group 1, Group 2 had a significantly higher association with the identification of possible underlying aetiology (16 vs. 4, *p* < 0.001) and a change in the management (10 vs. 3, *p* = 0.002).

**Conclusion:**

Diagnostic workup in patients with MINOCA was limited to CAG in 61.8% of patients in this study. However, patients with advanced workup had a significantly higher association with the change in the treatment and identifying possible underlying aetiology in such patients.

## Background

A substantial number of patients (5–10%) with acute myocardial infarction (MI) lack significant luminal stenosis on coronary angiography (CAG), thus warranting further workup to investigate the possible underlying aetiology [1–3]. Such patients fulfilling the universal definition of MI, along with nonobstructive coronary artery (< 50% stenosis) and without any other cause of acute presentation are referred to as myocardial infarction with nonobstructive coronary arteries (MINOCA) [1, 3, 4]. Its prevalence is variable (1–14%) based on the study population and even higher in autopsy-based studies [5–7]. This was previously considered a false positive MI before its recognition as a distinct entity comprising heterogeneous conditions [8]. Despite the recent shreds of evidence favoring the use of intravascular imaging such as optical coherence tomography (OCT), intravascular ultrasound (IVUS) and cardiac magnetic resonance imaging (CMR), many patients with MINOCA remain poorly worked up beyond CAG either due to limited resources and cost or lack of establishment of such practices in this relatively new entity where one diagnostic algorithm may not fit well to address this condition with heterogeneous aetiology [9–12]. Therefore, we conducted “the Evaluation of the clinical Profile, Investigations and Cardiac-Imaging of the patients with MINOCA (EPIC-MINOCA Study)” to assess whether patients with MINOCA benefits with advanced diagnostic workup beyond coronary angiography in this population.

## Methods

### Study design and settings

This single-center, observational study was conducted in a tertiary care center in North India after ethical clearance from the institutional ethical committee (AIIMS/IEC/22/246, May 2022) of AIIMS Rishikesh. This study was done from May 2022 to May 2023.

### Participants

**Fig. 1:**
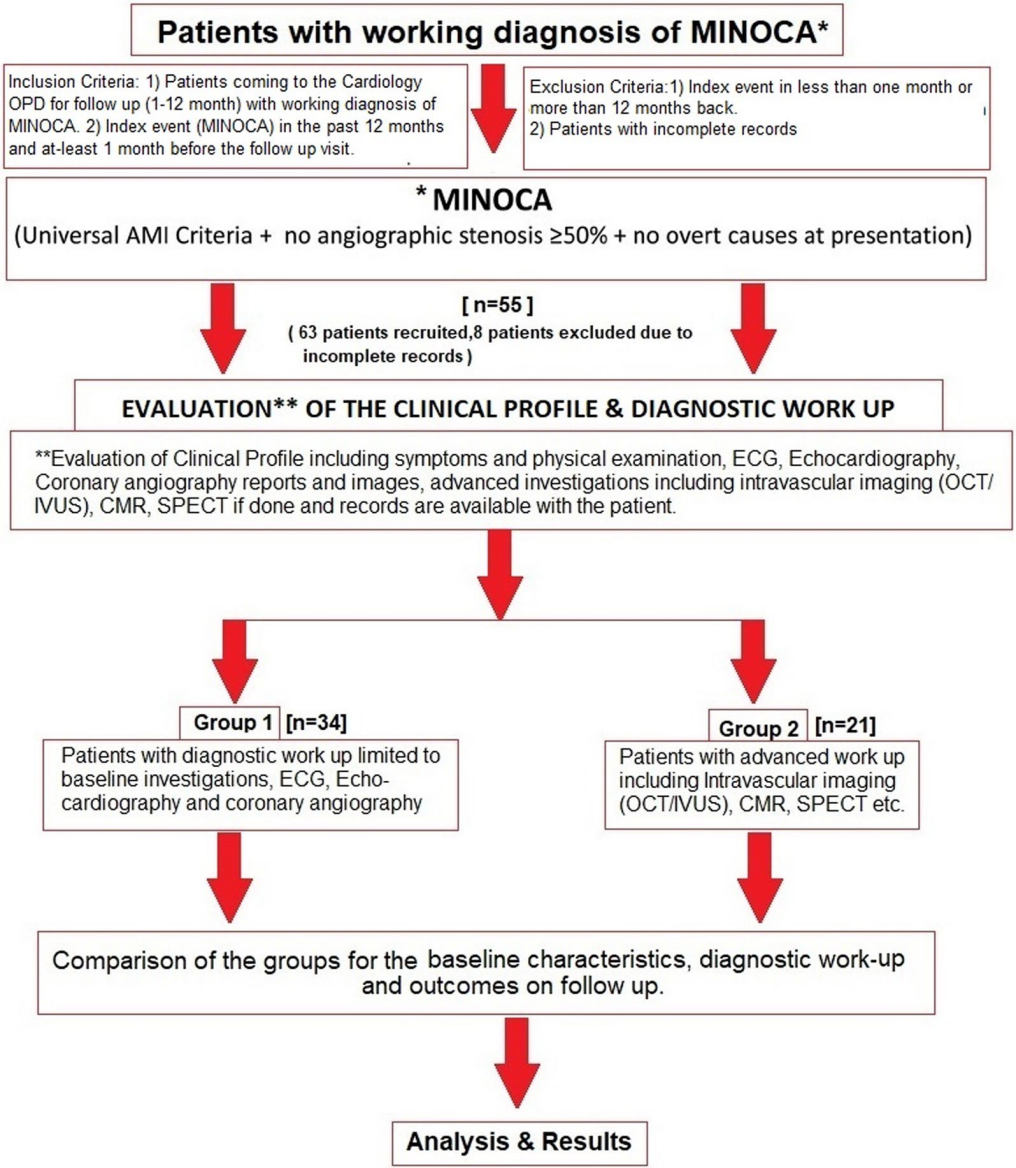
Study design and flow diagram

A total of 55 patients with a working diagnosis of MINOCA were recruited for the study at the time of their follow-up visit to the cardiology outpatient department (OPD) after obtaining written informed consent from all the participants. Those patients with index events within 30 days and beyond 12 months and patients with incomplete data were excluded from the study. Therefore, only patients with MINOCA after 1–12 months of index event were evaluated at the time of follow-up visit for the clinical characteristics, diagnostic workup including underlying aetiology (ischemic, nonischemic, spontaneous coronary artery dissection, slow flow, plaque disruption, etc.) and subsequent change in the management (after diagnostic workup). Inclusion and exclusion criteria along with the study design are summarised in Fig. 1.

### Variables

The primary variable was identifying the possible underlying aetiology in patients with MINOCA. Secondary variables were symptoms or CV events since index event and change in the management following the diagnostic workup.

### Data sources

All patients’ data were recorded in patient proforma including diagnostic workup since index event. All the patients with a working diagnosis of MINOCA were screened for the clinical profile, symptoms and physical examination records of index event. Baseline investigations of these patients including complete blood count (CBC), renal function test (RFT), lipid profile, HbA1C, electrocardiography (ECG), echocardiography, troponin-I report and CAG report along with images were evaluated for the presence of possible underlying aetiology of MINOCA. Patients who had other investigations including advanced diagnostic workups such as OCT, IVUS, CMR and nuclear imaging, i.e., single-photon emission computed tomography (SPECT) were evaluated for the presence of any significant finding. These patients were assessed at the time of the follow-up visit to the cardiology OPD for the evaluation of data from the index event till the current visit. The evaluation included assessment for the presence of any symptoms or CV events and change in the treatment after diagnostic workup. All the diagnostic workup record available with the patient was screened for all the relevant diagnostic tests and reports available with the patient to inspect any missed finding or other relevant finding. Patients based on these records were divided into two groups; Group 1 consisted of patients with limited diagnostic workup confined to CAG apart from baseline investigations including echocardiography (ECG) and blood tests which were available with all the patients. Group 2 consisted of patients with advanced workup beyond CAG such as OCT, IVUS, CMR or SPECT. A comparison of both the groups was done to evaluate if any significant difference exists.

### Statistical methods

The characteristics of the patients were summarised as mean with standard deviation (SD), median for troponin levels (due to skewed distribution) and percentages for categorical variables. The comparison was done using Chi-square tests (or Fisher's test if > 20% of expected cell counts < 5) for categorical variables and student t-tests (for continuous variables). A two-sided *p*-value of < 0.05 was considered statistically significant. All statistical analyses were performed using SPSS 25 (IBM Corp, Armonk, NY, USA).

## Results

### Baseline characteristics

A total of 55 patients with the working diagnosis MINOCA were evaluated in this study; their findings are summarised in Table 1.

**Table 1:** Clinical profile and investigations of patients with MINOCA

Parameters	*n* (%) or mean + SD
Age (Years)	48.6 ± 13.9
Male (*n*, %)	47 (85.5%%)
Smoker	27(49.1%)
*Comorbidities*	
Hypertension	18(67.3%%)
Diabetes	14 (25.5%)
CVA	1 (1.8%)
*Symptoms*	
Typical chest pain	52(94.5%)
Atypical chest pain	3 (5.5%)
Dyspnea	14 (25.5%)
Others	6 (10.9%)
*Laboratory parameters*	
Hemoglobin (g/dl)	14.3 ± 1.9
S. Urea (mg/dl)	29.7 ± 12.8
S. Creatinine (mg/dl)	0.9 ± 0.2
eGFR (ml/min/1.73m^2^)	103.0 ± 19.5
LDL (mg/dl)	93.4 ± 35.8
Peak Trop-I* in ng/ml (Median)	2.3
*ECG*	
ST elevation/Q (V2-V4)	12(21.8%)
Inferior wall MI	4(7.3%)
ST depression/ T inv/other QT prolongation	39 (70.9%) 14 (25.45%)
*Echocardiography*	
LVEF (%)	47.9 ± 11.0
LVEF ≤ 30%	3 (5.5%)

*Normal range of lab test used: 0.010–0.023 ng/ml. Lowest Trop-I: 0.34 ng/ml

#### Clinical profile

Patients were predominantly male (85.5%), with typical chest pain as the most common presentation (94.5%) accompanied by dyspnea in 25.5%. Diabetes and hypertension were the risk factors in 25.5% and 32.7% of the patients respectively while smoking in 49.1%.

#### ECG changes

ECG findings suggesting ST elevation or changes of evolved MI were evident in V2-V5/6 in 21.8% and in inferior leads in 7.3%. Other changes including T inversion, and ST depression were seen in the remaining 70.9% of the patients. QT prolongation was seen in 25.45% of patients.

#### Echocardiography findings

Mean left ventricular ejection fraction (LVEF) was 47.9 ± 11% with severe LV dysfunction (LVEF < 30%) found in 3 patients.

#### CAG

All patients had undergone conventional CAG and had normal epicardial coronaries in 29.1%, while in those with nonobstructive coronaries (69.9%); plaquing with luminal stenosis < 50% was the predominant finding followed by slow flow phenomenon (Table 2).

**Table 2:** Coronary angiography, intravascular imaging and cardiac imaging findings of patients with MINOCA

Investigation	n (%) or mean ± SD
Coronary angiography	55 (100%)
Normal	16 (29.1%)
Nonobstructive CAD	39 (69.9%)
Plaquing (< 50% stenosis)	30(54.5%)
Thrombus	3 (5.5%)
SCAD	2 (3.6%)
Slow flow	4 (7.3%)
OCT	7 (12.7%)
Plaque rupture	3 (5.5%)
Plaque erosion	1(1.8%)
Normal	3 (5.5%)
IVUS	4 (7.3%)
Plaquing+	3 (5.5%)
Normal	1(1.8%)
CMR	7 (12.7%)
LGE	3 (5.5%)
Normal	4 (7.3%)
SPECT	6 (10.9%)
Abnormal	5 (9.1%)
Normal	1(1.8%)

CAD, coronary artery disease; OCT, optical coherence tomography; IVUS, intravascular ultrasound; CMR, cardiac MRI, SPECT, single-photon emission computed tomography

### Advanced workup in patients with MINOCA

Only 21 patients had advanced workup in their records; in the remaining 34 patients, the workup was limited to CAG (Table 2).

#### Intravascular imaging

Of 11 patients having intravascular imaging records, 7 had undergone OCT, while 4 were investigated with IVUS (Table 2).

#### CMR

It was done in 7 patients, with 3 abnormal showing late gadolinium enhancement (LGE) and 4 with normal CMR. Of 3 abnormal CMR, one had ischemic type (subendocardial) LGE, while two had nonischemic (mid-myocardial) LGE.

#### Nuclear imaging

Cardiac SPECT was done in 6 patients with 5 showing evidence of ischemia, while 1 had normal findings.

### Aetiology and outcomes

**Fig. 2:**
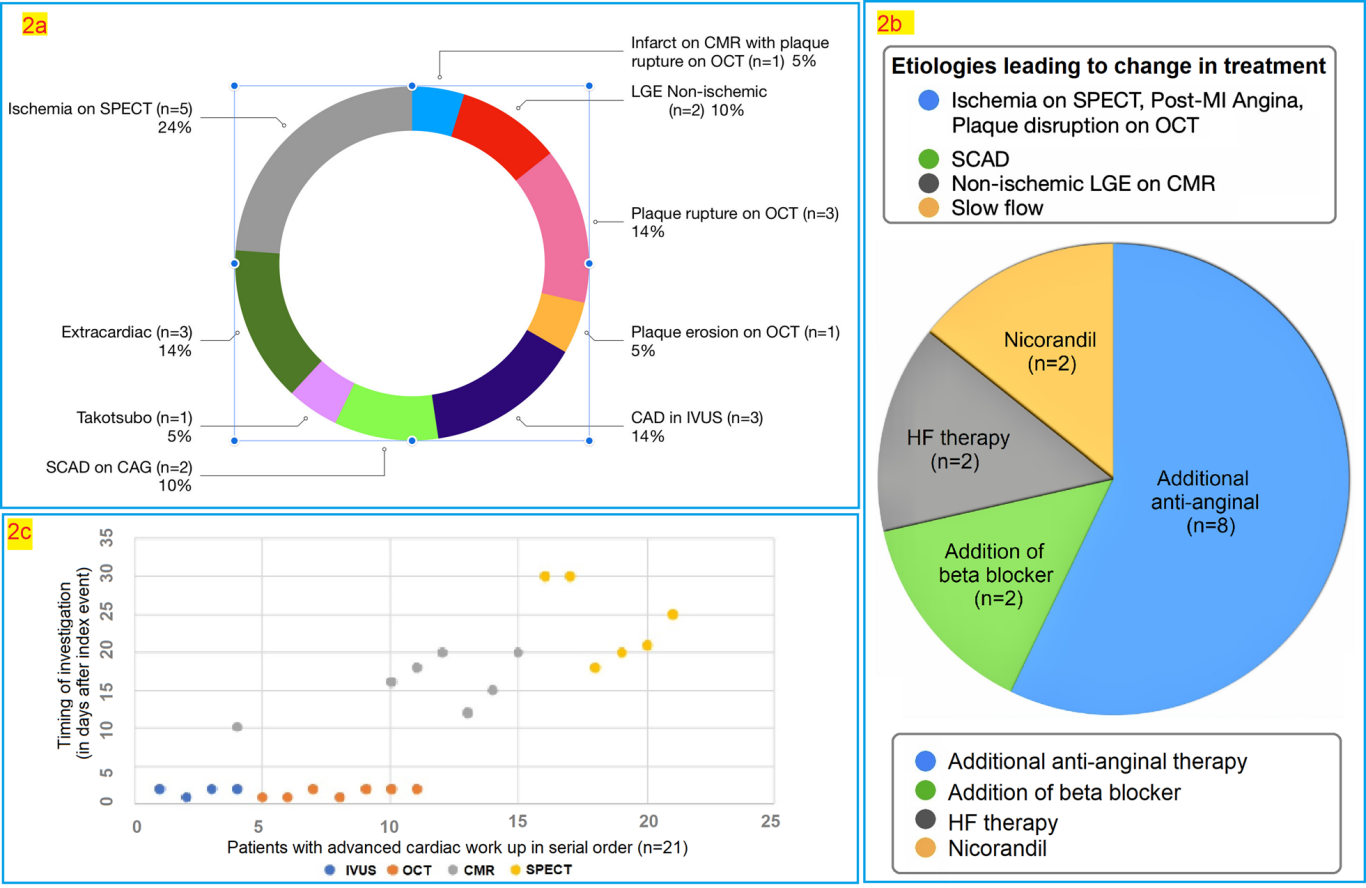
The spectrum of aetiology identified in patients with MINOCA after diagnostic workup (**2a**); Pie chart showing management/therapy added subsequent to findings on diagnostic workup. Findings/aetiology on diagnostic workup mentioned in the upper segment with the same color coding corresponding to the change in treatment depicted in the pie chart and mentioned in the lower segment (**2b**); scatter diagram depicting the number of days after index event in Y-axis and patients in X-axis with imaging modality in color-coded dots (**2c**)

A possible underlying aetiology was identified in 36.36% of patients (Fig. 2A). The time duration from the index event to the current evaluation was 3–6 months (mean 4.5 months). No mortality was reported in any patient from the index event till the current evaluation (mean duration 4.5 months). Change in the management occurred in 23.6% (13/55) of patients (Fig. 2B).

### Timing of diagnostic workup

Intravascular imaging was done within 2 days of the index event, while CMR and SPECT were done after 10–20 days (median = 16) and 18–30 days (median = 23) from the index event, respectively (Fig. 2C).

### Comparison of the patients with limited workup (Group 1) and advanced workup (Group 2)

**Fig. 3:**
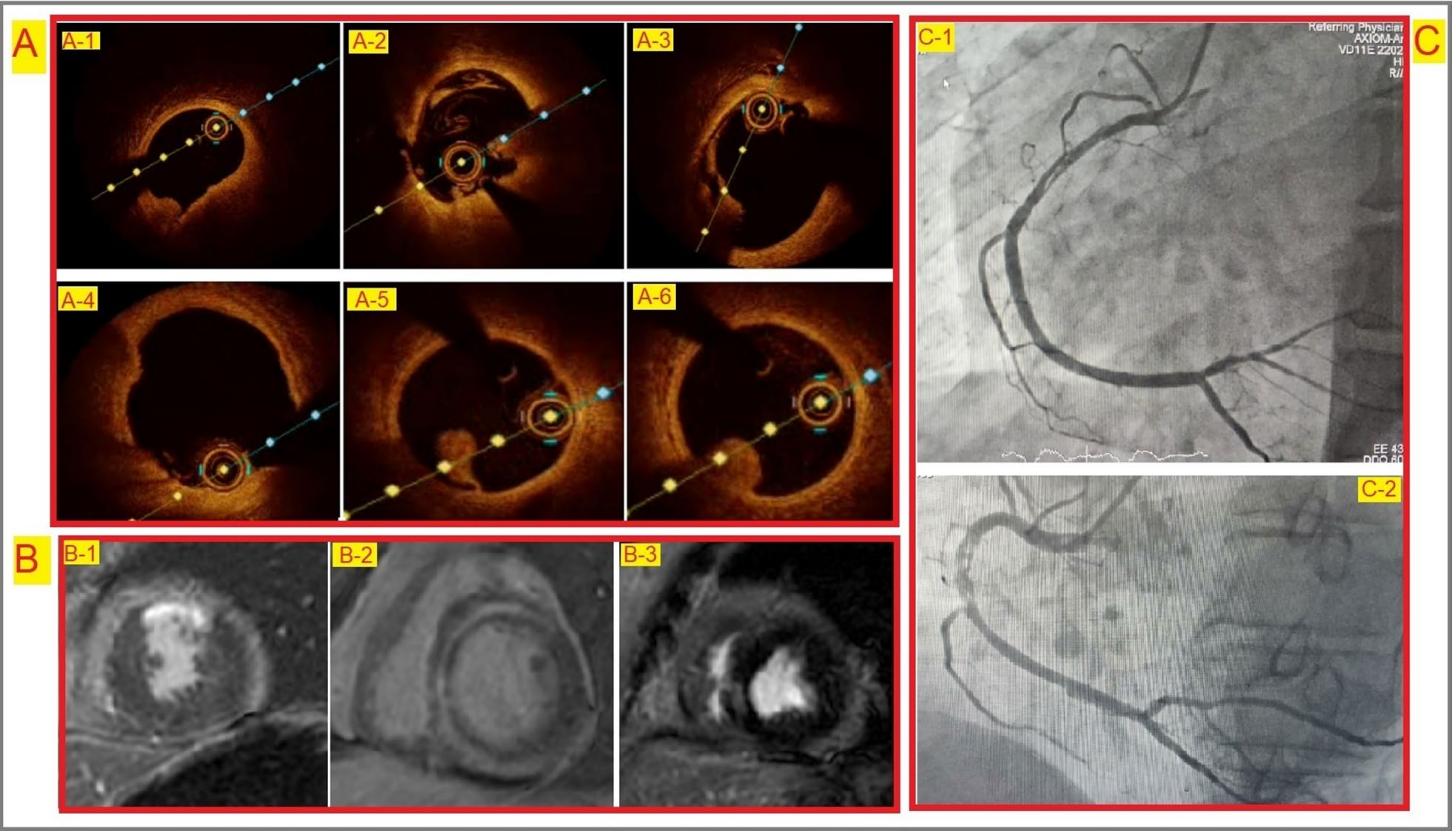
Multimodality imaging in patients with MINOCA. Images of OCT from different patients with MINOCA (Panel **A**), small plaque rupture (**A1**), plaque rupture with small white thrombi (**A2**), plaque rupture with thin cap (**A3**), plaque erosion with distal small thrombus (**A4**), white thrombus (**A5** and **A6**); Panel **A**. Images of CMR showing ischemic type (**B1**) and nonischemic type (**B2**, **B3**) LGE; Panel **B**. Images of CAG showing SCAD in right coronary artery (**C1**, **C2**); Panel **C**

On comparison, identification of possible underlying aetiology with 16 in Group 2 versus 4 in Group 1 (p < 0.001) and change in treatment, 10 in group 2 versus 3 in group 1 (*p* = 0.002) was found to be statistically significant (Table 3). Various distinct findings on imaging are shown in Fig. 3. Asymptomatic patients were predominant in Group 1 (31 vs. 15 in Group 2, p = 0.07); however, this difference was not statistically significant.

**Table 3:** Comparison between Group 1 and Group 2

Parameters	Group 1 (n = 34)	Group 2 (n = 21)	*p*-Value
Age (Years)	50.91 ± 11.9	45 ± 15.2	0.298
Male (*n*, %)	29 (85.2%)	18 (85.7%)	1.00
Smoker (*n*, %)	15 (44.1%)	12 (57.1%)	0.348
Hypertension (*n*, %)	14 (41.1%)	4 (19.0%)	0.089
Diabetes (*n*, %)	10 (29.4%)	4 (19.0%)	0.391
Typical chest pain	32 (94.1%)	20 (95.23%)	0.859
Dyspnea (*n*, %)	6 (17.6%)	8 (38.0%)	0.091
Hemoglobin (g/dl)	14.5 ± 2.0	14.1 ± 1.7	0.447
S. Urea (mg/dl)	29.4 ± 13.3	30.3 ± 12.4	0.816
S. Creatinine (mg/dl)	0.9 ± 0.2	0.9 ± 0.3	0.745
eGFR (ml/min/1.73m^2^)	97 ± 18.8	97 ± 21	0.708
LDL (mg/dl)	91.15 ± 34.1	﻿97﻿.14 ± 39.0	0.173
LVEF (%)	47.9 ± 10.3	46.9 ± 12.4	0.114
Symptomatic on follow-up (*n*, %)	3 (8.8﻿%)	6 (28.6﻿%)	0.071
Possible etiology identified (*n*, %)	4 (11.7%)	16 (76.2%)	< 0.001*
Change in the management (*n*, %)	3 (8.8%)	10 (47.6%)	0.002*

*p-Value of < 0.05 is considered significant; Group 1: MINOCA patients with limited diagnostic workup; Group 2: MINOCA patients with advanced diagnostic workup

#### Identification of possible aetiology and change in the treatment

In Group 1 (limited workup), 2 patients had SCAD; 1 had slow flow and another 1 patient had suspected takotsubo cardiomyopathy. Patients with SCAD had a change in the treatment with the addition of a beta-blocker; nicorandil was added to the patient with slow flow. One patient with suspected takotsubo cardiomyopathy was on follow-up without any change in the treatment.

In Group 2 (Advanced workup), 5 patients were positive for ischemia on SPECT, with one having slow flow on CAG requiring the addition of nicorandil; the remaining 4 were started on nitrates. Another patient with slow flow had an additional finding of plaque erosion on OCT and was kept on a high-intensity statin with dual antiplatelet therapy (DAPT) along with nicorandil. Three patients with plaque rupture on OCT were also kept on high-intensity statin, DAPT and additional antianginal therapy (nitrates and beta-blockers). Out of these 3, one had infarct on CMR and was already on diuretics and other HF therapy; the remaining 2 had nonischemic LGE in CMR requiring a change in treatment with the intensification of HF therapy (Fig. 2a, b). All the patients in both groups received DAPT, statins and heparin (at least for an initial 48 h). Patients with LV dysfunction received guideline-directed medical therapy for heart failure and required change in the HF therapy (with the use of angiotensin receptor/neprilysin inhibitor in place of Ramipril and introduction of empagliflozin) in two patients with CMR finding of nonischemic LGE.

## Discussion

The results of this study showed that less than two-fifths of the patients with MINOCA were dealt with advanced diagnostic workup; however, such patients with advanced workup had significant differences in terms of identification of possible aetiology of MINOCA and change in treatment when compared to the patient with limited diagnostic workup.

The clinical profile of the patients in this study comprises predominantly males in contrast to the other studies having slightly higher representation of women despite male predominance [2, 7, 12]. Although studies from this subcontinent are limited, most of the other studies are based on retrospective registry-based data; data for this study were collected at the time of the follow-up visit with a retrospective evaluation of the data available with the patient from the index event till the current visit. A systematic review of 28 publications on MINOCA by Pasupathy et al. found a mean age of 58.8 years with 43% being women, in which hypertension was the predominant risk factor in 52%, diabetes in 15% and smoking in 42% of the patients, which is nearly similar to the results of this study apart from male predominance and a relatively younger population in the present study (mean age = 48.6 years) [2, 12, 13]. The presence of traditional risk factors of CAD (diabetes, hypertension, smoking) is less frequent in patients with MINOCA when compared with MI-CAD patients although it varies across different studies [2, 7, 10, 12, 13].

The most common presentation of patients in this study was typical chest pain (94.5%) accompanied by dyspnea in 25.5%; however, symptomatology in MINOCA is not well discussed in the currently available literature. ECG findings suggest NSTEMI was predominant followed by AWMI, similar to the other studies with NSTEMI being the most common presentation in MINOCA [1, 2, 14].

In this study, the median troponin level was 2.9 ng/ml, with the lowest value of 0.34 ng/ml. A study by Williams et al. [15] evaluating the yield of CMR in 719 patients with MINOCA found a peak troponin threshold of 211 ng/L (equivalent to 0.211 ng/ml) as optimal. All the patients in this study had higher troponin levels than this cutoff.

CAG revealed normal epicardial coronaries in 29.1% of the patients in the present study; however, this is as high as 51% in other studies [1, 2, 14], Prognostic difference in outcomes in smooth coronary versus some irregularity in coronary was evaluated in very few studies with poorer outcomes in the former evident in one such study with a much smaller sample size [16]. No mortality was reported in the present study. Of those with nonobstructive coronaries, plaquing with luminal stenosis < 50% was predominant in this study. Other findings were slow flow, thrombus and spontaneous coronary artery dissection (SCAD). Provocative spasm testing was not done in any of these patients, thus coronary spasm as a potential mechanism might have been unmasked. Approximately one-fourth of patients with MINOCA and 20–80% of MI-related CAD (MI-CAD) are reported to have inducible coronary spasms, and these data vary widely across different studies [17–19].

Advanced workup including OCT, IVUS, CMR and SPECT was done on ~ 38.2% of patients in this study. OCT (*n* = 7), could reveal abnormality in 4 patients (3 with plaque rupture and 1 with plaque erosion). In one such study, OCT detected thrombi or plaque disruption in 39% of the patients (*n* = 38), while in another study evaluating 27 patients, 78% of them had either plaque disruption or thrombi on OCT imaging [20, 21]. In a study by Gerbaud et al. [22] evaluating 40 patients with MINOCA, plaque rupture and plaque erosion was evident on OCT in 14 (35%) and 12 (30%) patients, respectively. Certain drawbacks exist with OCT as findings in the left main ostium might be missed; however, OCT has been largely advocated for the use in MINOCA with the emerging evidence in the recent studies [20, 23, 24]. However, its clinical implications in long-term follow-up need to be evaluated.

IVUS for MINOCA has been used less commonly than OCT [23, 24]. As compared to OCT, IVUS does not require additional contrast injections and, therefore findings such as SCAD, dissections, small thrombi and plaque can also be assessed with IVUS in patients with MINOCA [24, 25]. In one such study, plaque disruption was identified in 38% (16/42) of women with MINOCA [26]. In the present study, IVUS was done in 4 patients with 3 having plaquing; however, plaque disruption was not identified in any of these patients, while 1 had normal findings. Although OCT has better tissue resolution, it is not widely available and therefore IVUS can be useful in identifying the cause wherever intravascular imaging is indicated as per clinical scenario. However, studies are limited in this context.

CMR is emerging as an important diagnostic tool in patients with MINOCA. It provides evidence of MI if done timely (preferably within 2 weeks) and more importantly it provides important clues in diagnosing several other conditions such as cardiomyopathy, myocarditis, takotsubo cardiomyopathy, etc. [5, 24, 26]. In this study, CMR was done in 7 patients, of which 3 had LGE, while 4 were normal. However, the timing of CMR in this study with a median duration of 16 days from the index event, is slightly delayed than other studies. CMR is preferred within 2 weeks of the index event in patients with MINOCA [5, 26]. A study by Opolski et al. found LGE in 52% (16/31) of patients with MINOCA undergoing CMR [21, 27]. In a similar study by Gerbaud et al. evaluating CMR in patients with MINOCA; evidence of MI was evident in 77.5% (31/40) of the patients [22, 27]. Abnormal CMR with LGE in 74.1% (86/116) of patients with MINOCA with ischemic LGE pattern in 53.4%(62/116) was evidenced in another such study [26, 28]. In CMR, it is important to distinguish LGE into ischemic (subendocardial/transmural) versus nonischemic (mid-myocardial LGE/epicardial) in patients with MINOCA, as the former provides evidence of MI; the latter conditions suggest the possibility of conditions mimicking MI such as myocarditis [28, 29]. In the present study, only 1 patient had subendocardial LGE, while two had mid-myocardial LGE.

The use of SPECT in MINOCA has been evaluated in fewer studies. One such study evaluating SPECT in the absence of obstructive disease suggested its usefulness in relevance to microvascular dysfunction [30]. Another study showed that SPECT in such patients correlated with the transmural extent of MI on CMR [31]. Microvascular dysfunction is considered an important cause of MINOCA; however, the role of cardiac imaging to evaluate the same remains limited [32]. In this study, 6 underwent SPECT; of 6 patients, 5 had ischemia evidenced on SPECT.

Outcomes in patients with MINOCA were considered good previously; however, the 1-year all-cause mortality rate is ~ 3.5% (1.15–3.5%) in such patients [33–35]. In the present study, no mortality was reported, although 16.36% (9/55) patients were symptomatic on follow-up. In another study, a 23.9% rate of major cardiac events (MACE) were reported during the mean follow-up period of 4.1 years [2, 36]. Thus, the low sample size and limited follow-up data might be responsible for low mortality in this study. Moreover, aetiology-based mortality data in MINOCA is limited [33–36]. Another finding of a higher number of asymptomatic patients in group 1 (31 vs. 15 in group 2, *p* = 0.07) might reflect the tendency of the symptomatic patients to opt for advanced cardiac workup after nonobstructive epicardial coronaries on CAG. However, longer follow-up data can better reveal the underlying aetiology (if any).

The identification of possible underlying aetiology in patients with MINOCA is difficult and depends on early diagnostic workup. In a study by Reynolds et al., the cause was identified in 84.5% (98/116) after evaluating both CMR and OCT in 116 patients with MINOCA [26, 37]. In the present study, possible underlying aetiology was identified in 36.3% (20/55) patients. Change in the management was noticed in 13 patients in the present study. Both the parameters, i.e., change in management and identification of possible aetiology had significant association with patients who underwent advanced diagnostic workup. However, the role of change in management strategy (including pharmacological therapy based on underlying aetiology) in long-term outcomes in MINOCA is limited [38]. A lower risk of recurrent SCAD with a beta-blocker has been reported in one study [38]. Nitrates can provide symptomatic benefit in coronary spasms. A meta-analysis showed a favorable hazard ratio with statins or angiotensin-convertase enzyme inhibitors (ACEi)/Angiotensin receptor blockers (ARB) in reducing major adverse cardiovascular events (MACE) in patients with MINOCA [36, 39, 40]. Results of MINOCA-BAT study and BA-SCAD study can unveil the role of pharmacotherapy in MINOCA [2, 40].

This study evaluated the clinical profile and diagnostic workup in patients with MINOCA and showed that diagnostic workup in ~ 61.8% of patients with MINOCA was limited to CAG. Advanced diagnostic workup is limited to less than two-fifths of the patients with MINOCA in this study. However, patients with advanced workups had a significant association with the identification of possible underlying aetiology and subsequent change in the treatment in these patients, in contrast to patients with diagnostic workups limited to CAG. This study shows the role of advanced workup in patients with MINOCA and therefore paves the way for future research in this context. Moreover, this study addresses the need for comprehensive diagnostic workup in patients with MINOCA.

## Limitations

The small sample size is the limitation of this study. Late execution of CMR was another drawback as early CMR might have unmasked certain other etiologies in these patients. A well-designed trial with long follow-up data for the outcomes could better highlight the role of advanced imaging in patients with MINOCA.

## Conclusions

Patients with MINOCA evaluated with advanced work (OCT/IVUS, CMR or SPECT) had a significant association with the identification of possible underlying aetiology and subsequent change in the management. However, evaluation with advanced workup was limited to less than two-fifths of the patients in this study.

## Data Availability

The datasets used and analyzed during the current study are available from the author on reasonable request.

## Abbreviations

ACEI: Angiotensin-convertase enzyme inhibitors
ARB: Angiotensin receptor blockers
AWMI: Anterior wall myocardial infarction
CAD: Coronary artery disease
CAG: Coronary angiography
CBC: Complete blood count
CMR: Cardiac magnetic resonance imaging
ECG: Electrocardiography (ECG)
IVUS: Intravascular ultrasound
IWMI: Inferior wall myocardial infarction
LGE: Late gadolinium enhancement
LVEF: Left ventricular ejection fraction
MI: Myocardial infarction
MINOCA: Myocardial infarction with nonobstructive coronary arteries
OCT: Optical coherence tomography
OPD: Outpatient department
RFT: Renal function test
SCAD: Spontaneous coronary artery dissection
SD: Standard deviation
SPECT: Single-photon emission computed tomography
